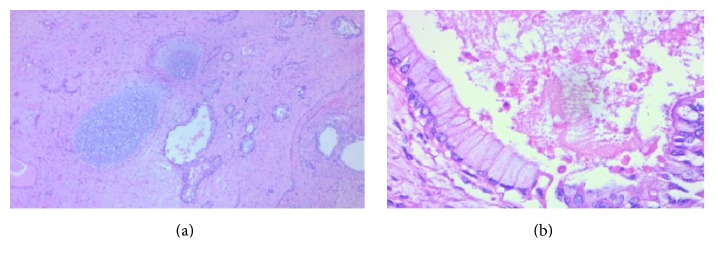# Testicular Cancer Presenting as Gastric Variceal Hemorrhage

**DOI:** 10.1155/2017/4510387

**Published:** 2017-11-06

**Authors:** Carlos Eduardo Salazar-Mejía, David Hernández-Barajas, Edio Llerena-Hernández, José Luis González-Vela, María Inés Contreras-Salcido, Adriana González-Gutiérrez, Omar David Borjas-Almaguer, Luis Alberto Pérez-Arredondo, Blanca Otilia Wimer-Castillo

**Affiliations:** ^1^Department of Internal Medicine, University Hospital “Dr. José Eleuterio González” and Faculty of Medicine, Universidad Autónoma de Nuevo León, Monterrey, NL, Mexico; ^2^Oncology Service and Department of Internal Medicine, University Hospital “Dr. José Eleuterio González” and Faculty of Medicine, Universidad Autónoma de Nuevo León, Monterrey, NL, Mexico; ^3^Gastroenterology Service and Department of Internal Medicine, University Hospital “Dr. José Eleuterio González” and Faculty of Medicine, Universidad Autónoma de Nuevo León, Monterrey, NL, Mexico

## Abstract

Testicular cancer is the most common solid malignancy affecting males between the ages of 15 and 35. The symptomatology caused by this tumor varies according to the site of metastasis. We present the case of a 26-year-old male who arrived to the emergency department with hematemesis. He had no previous medical history. On arrival, we noted enlargement of the left scrotal sac. There was also a mass in the left scrotum which provoked displacement of the penis and right testis. The serum alpha-fetoprotein level was 17,090 ng/mL, lactate dehydrogenase was 1480 U/L, and human chorionic gonadotropin was 287.4 IU/mL. Upper endoscopy revealed a type 1 isolated gastric varix, treated with cyanoacrylate. A CT scan showed extrinsic compression of the portal vein by lymphadenopathy along with splenic vein partial thrombosis, which caused left-sided portal hypertension. Neoadjuvant chemotherapy was started with etoposide and cisplatin, and seven days later the patient underwent left radical orchiectomy. A postoperative biopsy revealed a pure testicular teratoma. Noncirrhotic left portal hypertension with bleeding from an isolated gastric varix secondary to metastasic testicular cancer has not been described before. Clinicians must consider the possibility of malignancy in the differential diagnosis of a young man presenting with unexplained gastrointestinal bleeding.

## 1. Introduction

Testicular cancer is the most common solid malignancy affecting men between the ages of 15 and 35 [1]. The symptomatology caused by this tumor varies according to the site of metastasis. We present a rare case of nonseminomatous germ cell testicular cancer with hematemesis as the presenting symptom.

## 2. Case Report

A 26-year-old man arrived at the emergency department with a seven-day history of hematemesis and melena. He had no previous medical history and did not drink alcohol or used any illicit drug or medication.

Physical examination on admission showed blood pressure of 100/60 mmHg, a temperature of 36°C (96.8°F), a pulse rate of 90/min, and a respiratory rate of 22/min; his height was 1.75 m, weight 98 kg, and BMI 32 kg/m^2^. He experienced pain with deep palpation in the epigastrium and no organomegaly or lymphadenopathy was identified. The left scrotal sac was enlarged and indurated and there was a mass in the left scrotum that was indistinguishable from the left testis and provoked displacement of structures of the penis and right testis (the right scrotum was empty). No inguinal lymphadenopathy was identified.

Laboratory tests revealed normal liver function. Hemoglobin was 9.13 g/dl, MCV was 87.9 fL, WBC was 11.9 K/uL, neutrophils were 9.81 K/uL, lymphocyte count was 1.59 K/uL, and platelet level was 252 K/uL. Serum glucose level was 106 mg/dl, BUN was 38 mg/dl, creatinine was 0.9 mg/dl, and calcium was 8.9 mg/dl. Serum alpha-fetoprotein (AFP) level was 17,090 ng/mL, lactate dehydrogenase was 1480 U/L (normal range: 91–180 IU/L), and human chorionic gonadotropin level was 287.4 IU/mL.

**Figure 1:**
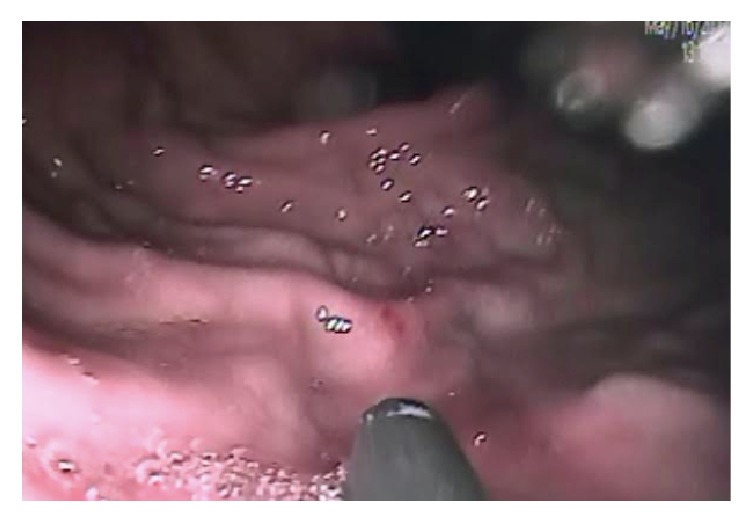


After resuscitation with crystalloid solutions, he underwent upper endoscopy. The gastroenterologist found abundant active bleeding, for which orotracheal intubation was decided to provide airway protection. The patient was transferred to the intensive care unit. A second upper endoscopy revealed a type 1 isolated gastric varix, treated with cyanoacrylate without complications (Figure 1).

A scrotal US showed a large heterogeneous image in the left testicle area, with an echogenic and cystic solid component and flow presence with color Doppler assessment.

**Figure 2:**
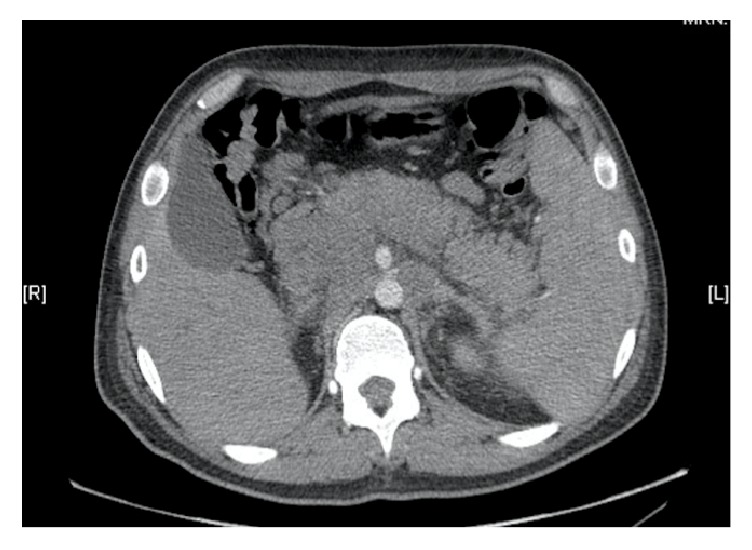


A contrasted CT scan of the thorax, abdomen, and pelvis showed a liver of normal size and density with multiple retrocrural, retroperitoneal, mesenteric, and left iliac metastatic lymphadenopathy which caused extrinsic compression of the portal vein along with splenic vein partial thrombosis with left-sided portal hypertension and perigastric and perisplenic collateral neovascularization (Figure 2). A heterogeneous, well defined mass was found in the left testicle, 16.7 × 16.1 × 14.9 cm, with a solid component that was enhanced with the administration of contrast, as well as a cystic component. There was also invasion of the left spermatic cord.

The patient was extubated after surveillance and transferred to the Internal Medicine Department. We started chemotherapy with etoposide 100 mg/m^2^ and cisplatin 20 mg/m^2^, and seven days later the patient underwent a left radical orchiectomy by an inguinal approach with left hemiscrotectomy, without complications. Following this intervention, serum alpha-fetoprotein (AFP) level was reduced to 350 ng/mL, and the human chorionic gonadotropin level was 50 IU/mL.

A postoperative biopsy showed a pure testicular teratoma (Figure 3) with glandular formations and the presence of cartilage (a) and respiratory epithelium, with ciliated columnar cells alternating with goblet cells (b).

The patient was discharged after showing clinical improvement to receive ambulatory chemotherapy.

## 3. Discussion

Germ cell tumors account for 95 percent of all cases and are divided into two groups: seminomas and nonseminomas [1]. Pure testicular teratoma is rare, accounting for only 2 to 6% of all primary testicular tumors. Despite its histologically benign appearance, the clinical course of primary pure teratoma is unpredictable [2].

A painless testicular mass in a young man is pathognomonic of testicular cancer; nevertheless, the majority of cases present with diffuse testicular pain, swelling, and/or hardness [3]. The rest of the manifestations are attributable to metastatic disease; symptoms vary with the site of metastasis: a neck mass due to supraclavicular node metastasis, cough or dyspnea with pulmonary metastasis, bone pain with skeletal metastasis, and central or peripheral neuropathy with nervous system involvement.

Less than 5% of patients with testicular cancer present with gastrointestinal involvement [4]. This is mainly associated with metastatic disease including the duodenum, jejunum, ileum, stomach, esophagus, colon, or pancreas.

There are reported cases of gastrointestinal bleeding associated with metastatic testicular cancer with intestinal involvement, which is a rare manifestation of the disease [5–7]. However, noncirrhotic left portal hypertension due to extrinsic compression of the portal vein and partial thrombosis of the splenic vein, with bleeding from an isolated gastric varix secondary to testicular cancer, has not been described before [8].

Left-sided portal hypertension accounts for less than 5% of all patients with portal hypertension [9], and pancreatic disorders are the most frequent cause [10]. Gastric variceal bleeding generally tends to be more severe [11], and treatment with cyanoacrylate usually provides hemostasis in 80–90%. Surgery is reserved for refractory patients [10, 12].

Teratoma represents 4% of all testicular germ cell tumors. The age of presentation is about 20–40 years. It originates from malignant germ cells and has 12p isochromosome amplification. More than 90% show IGCNU and cytologic atypia. It presents with nonteratomatous metastases in 20–40% of cases. There are malignant gonadal teratomas that are malignant because of their derivation from a malignant germ cell through intermediary forms of an invasive germ cell tumor, such as yolk sac tumor or embryonal carcinoma. This applies to most postpubertal testicular teratomas [13].

The standard treatment for all testicular tumors in adults is radical inguinal orchiectomy. In the setting of a pure teratoma with advanced disease and elevation of tumor markers, chemotherapy is the systemic therapy of choice [14]. Approximately 70% to 80% of patients with metastatic disease will be cured with cisplatin-based chemotherapy combined with surgery to resect residual disease as an integral part of management. The therapeutic objective is cure, with distinct approaches for disease deemed good risk (high probability of cure) and poor risk (lower probability of cure) [15].

For 20% to 30% of patients with advanced germ cell tumors, their disease will fail to achieve a durable response to chemotherapy regimens, including cisplatin and etoposide with or without bleomycin. The combination of paclitaxel, ifosfamide, and cisplatin (TIP) was evaluated as second-line therapy for patients with favorable prognostic features for response, including testis primary tumor site and a prior complete response to first-line chemotherapy [16].

Our patient was unaware of the risk that carries the presence of a testicular mass. He never sought medical counseling after 10 months of evolution of the disease and did not even mention it as an antecedent at his arrival. Hence, the clinician must consider the possibility of malignancy in the differential diagnosis of a young man presenting with unexplained gastrointestinal bleeding or intestinal obstruction. It is also necessary to implement educational programs, in order to raise public awareness about testicular cancer.

## Figures and Tables

**Figure 3:**